# Experimental and Numerical Study to Enhance Granule Control and Quality Predictions in Pharmaceutical Granulations

**DOI:** 10.3390/pharmaceutics17030364

**Published:** 2025-03-13

**Authors:** Maroua Rouabah, Inès Esma Achouri, Sandrine Bourgeois, Stéphanie Briançon, Claudia Cogné

**Affiliations:** 1 Group of Research on Technologies and Processes GRTP, Université de Sherbrooke, Sherbrooke, QC J1N 0J8, Canada; 2 Laboratoire d’Automatique, de Génie des Procédés et de Génie Pharmaceutique (LAGEPP), UMR CNRS 5007, Université de Lyon, Université Claude Bernard Lyon 1, Domaine de la Doua, 69616 Villeurbanne, France

**Keywords:** numerical granulation, high-shear granulator, modeling, discrete element method, particle size distribution, scale-up study, pharmaceutical manufacturing

## Abstract

**Background/Objectives:** The pharmaceutical industry demands stringent regulation of product characteristics and strives to ensure the reproducibility of granules manufactured via the wet granulation process. A systematic model employing the discrete element method (DEM) was developed herein to gain insights into and better control this process. **Methods**: The model comprehensively simulates particle behavior during granulation by considering the intrinsic properties of the powder material, the specific geometry of the granulation equipment, and various operational conditions, including impeller speed and chopper use. Notably, this approach can simulate dynamic interactions among particles and integrate complex phenomena, such as cohesion, which is crucial for predicting the formation and quality of granules. **Results**: To further support process optimization, an EDEMpy artificial intelligence (AI) tool was developed as a posttreatment routine to monitor and analyze agglomerate size distributions, proving essential for assessing the efficiency of the granulation process and the quality of resulting granules. The DEM model was evaluated by comparing its output with experimental data collected from a 0.5 L high-shear granulator. The model reproduced the granule growth kinetics observed experimentally, confirming the agreement between the experimental and numerical analyses. **Conclusions**: This underscores the model’s potential in predicting and controlling granule quality in wet granulation processes, enhancing the precision and efficiency of pharmaceutical manufacturing.

## 1. Introduction

In the context of powder handling and processing, high-shear wet granulation is a prominent technique widely employed across diverse industries, including pharmaceutical, chemical, and food sectors. This process involves the adhesion of primary powder particles to form larger multiparticle entities known as granules, typically to enhance mechanical and flow properties. Recent research has highlighted the complex nature of the wet granulation process, summarized in three main points:

- The mechanisms of wet agglomeration can be defined as the succession of three stages: (i) wetting and nucleation; (ii) coalescence, consolidation, and growth; and (iii) rupture and attrition. Predicting the dominance of one mechanism over others is challenging, with most studies focusing on a single predominant mechanism [1]. Advances in the field have emphasized the complexity of these mechanisms and their interactions with material properties and granulation kinetics, especially concerning binder interactions and granule formation dynamics [2].
- Systematic studies have established links between operational parameters (such as filling rate and stirring speed, etc.), physicochemical characteristics of the binder (like wettability, angle of contact, and viscosity, etc.), and the quality of the final product (e.g., size distribution and flowability) [2,3,4,5,6]. However, the influence of these parameters on granule growth mechanisms varies with the raw materials and technologies employed, making it difficult to generalize the observed trends and hindering the overall optimization of the process.
- Online monitoring studies are implemented to assess material homogeneity during production, yet the dispersed nature of the solid phase complicates observations. Thus, monitoring is often limited to measuring torque on the stirring shaft, reflecting changes in powder consistency [5,6,7].

Predictive tools are crucial for identifying potential correlations between the intrinsic characteristics of the powder, the operational parameters, and the quality of the resulting granules. The extensive array of process parameters inherent in experimental studies necessitates the use of numerical simulations for effective process facilitation. A comprehensive bibliographic analysis in this study explores four types of models: (i) dimensional analysis, (ii) population balances, (iii) discrete element method (DEM), and (iv) coupled models. Dimensional analysis is primarily utilized for scaling up in high-shear granulation, considering factors like impeller speed and binder addition rate. Common dimensionless numbers used to describe the process and interactions at different scales include Newton, Froude, and Reynolds numbers [8]. Dimensional analysis has proven its relevance in scaling from a small-scale granulator (250 mL) to a larger scale [9]. Additionally, there exist dimensionless numbers that are instrumental to the characterization of growth and consolidation. These numbers are contingent upon operating parameters such as the spray flow and the liquid-to-solid mass ratio [10,11,12]. While this methodology simplifies implementation, it does not provide detailed information at the particle level, such as particle size evolution over time, critical for identifying key process parameters.

Population balance modeling (PBM) predicts granule growth and analyzes related mechanisms, aiming to elucidate temporal changes in granule properties [13,14]. These models are based on the conservation of numbers, monitoring particles with specific properties like size, porosity, and shape, and accounting for changes during growth, rupture, or other phenomena. Ramkrishna and Mahoney have detailed the general population balance equation [15]. The resolution can range from one-dimensional (focusing solely on diameter) to multi-dimensional, tracking additional properties like liquid binder content and porosity [16,17,18]. Multidimensional balances provide deeper insights into consolidation processes but lack details on physical particle collisions, such as collision counts, relative velocities, and impact energy, which significantly influence consolidation and rupture within the granulation vessel.

The discrete element method (DEM) is a numerical technique that accurately describes the discontinuous phase of a granular medium; it considers the geometry, the behavior of the component elements of the medium, and the laws of interaction between them. The method was originally proposed by Cundall and Strack, who posited that the behavior of a granular medium can be predicted if the movement of individual particles can be modeled [19]. The method is predicated on the interactions between a finite number of particles in contact with each other, incorporating contact models and the individual movements of each particle. This granular approach captures the unique dynamics of particles, including collisions, frictions, and adhesion, critical for understanding complex behaviors in granular assemblies. This theoretical framework enables one to determine the position and velocity of a given solid particle as the temporal solution of Newton’s second law for translation and rotation movements (balance of quantity of motion). The incorporation of transfer equations allows one to consider the physical, thermal, or chemical state of each particle [20]. The Hertz–Mindlin model, developed by Mindlin [21], is fundamentally employed for contact models; it delineates a relationship between the normal force of particles and normal overlap at the contact interface between two spheres [22]. The model can be further enhanced by incorporating the concept of particle cohesion. The most commonly employed model is based on the Johnson–Kendall–Roberts (JKR) theory [23]. Additionally, models of liquid bridges, incorporating a force of attraction stemming from surface tension and hydrostatic pressure, have been included [24]. These detailed contact interactions are critical for accurately simulating the behavior of granules during processing, impacting both the structure and dynamics of the material. A substantial corpus of research has employed the DEM to elucidate the granulation process, and applications have focused on the analysis and design of typical granulators [25,26,27]. The primary challenge of this study involves the validation of the model. Further, a comprehensive bibliographic analysis revealed a paucity of studies successfully integrating experimental and numerical methodologies.

Nevertheless, there are some hybrid models coupling DEM with other models, such as computational fluid dynamics or population balances [28,29,30,31]. These models have the capacity to simulate dynamics and complex mechanics; however, they present significant challenges when implemented in routine controls.

This study aims to develop predictive methodologies to better understand the links that may exist between the intrinsic characteristics of a powder, the operating parameters of granulations, and the temporal evolution of the quality of the produced granules. The model is solved by the DEM, and the tendencies are validated against experimental results.

## 2. Materials and Methods

### 2.1. Experimental Study

#### 2.1.1. Granulation Protocol

Microcrystalline cellulose (MCC; Vivapur^®^, spheres, grade 100, 70–140 mesh, 100–200 µm, JRS) was selected for the experimental production of granules. MCC of this specific grade and form was chosen despite the availability of other grades from the manufacturer due to its spherical form, which eliminated the variable of particle shape in our numerical study and allowed for the most realistic representation of the experimental conditions. Granulations of MCC were conducted using a high-shear laboratory wet granulator, the Glatt TMG 1/6, which is specifically designed for small-scale, research-focused granulation processes. This machine is equipped with a 0.5 L vessel, ideal for handling small batch sizes up to 61 g and allowing precise control over granulation parameters. It features an adjustable impeller and chopper speed, ranging from 100 to 3000 rpm, which facilitates the effective mixing, granulation, and densification of powdery substances. The protocol included dry premixing for 2 min at 100 rpm using only the impeller, followed by the drip spraying of a binder—a 3% *w*/*v* aqueous solution of polyvinylpyrrolidone (PVP K30) from BASF. Approximately 10 mL of the binder was utilized, with a feed rate of 1 mL/min implemented via a peristaltic pump. Granulation was continued for 10 min with an impeller speed of 600 or 900 rpm and a cross-blade chopper speed of 600 rpm, followed by maturation for 5 min with the batch size maintained at 61 g, as noted earlier. After granulation, the granules were subjected to processing following a 48 h air-drying period. The speeds of 600 and 900 rpm were selected as they ensure the ideal compromise between efficient mixing and desirable granule quality for our granulations. The moisture content of a sample of the granules was estimated at 3.02% after 6 min of analysis using a moisture analyzer (Sartorius MA37, Oakville, ON, Canada) at 120 °C under standard drying conditions. Both experimental granulation and moisture content tests have been conducted with a repeatability factor of 3. An experimental study on granulation in a 4 L bowl has been previously validated [32], and the subsequent results will be used to validate the scale-up study.

#### 2.1.2. Particle and Granule Characterization

**Figure 1:**
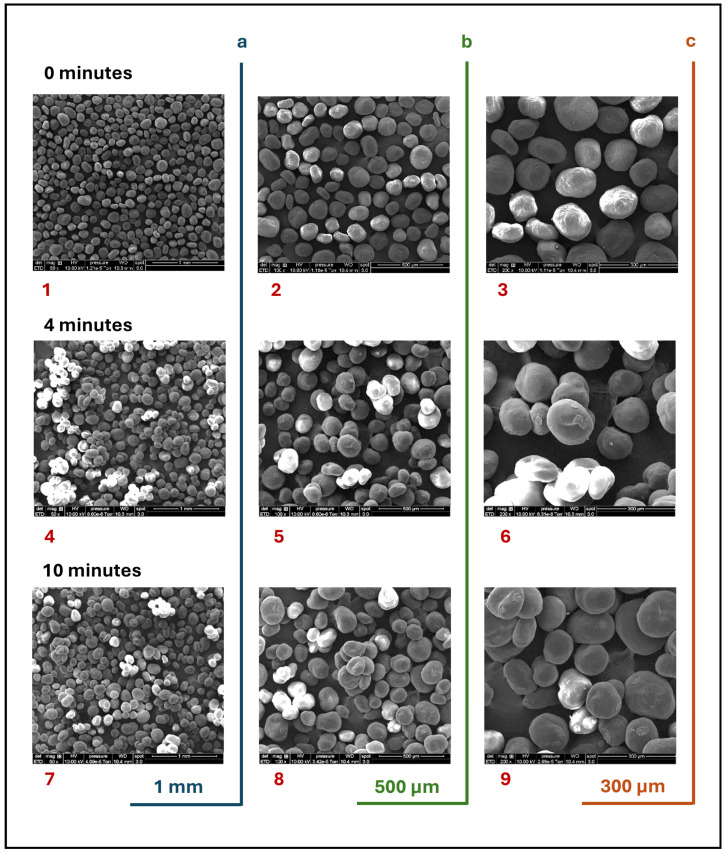
Scanning electron microscopy (SEM) images of granule formation over time for the 0.5 L vessel at 600 rpm.

**Figure 2:**
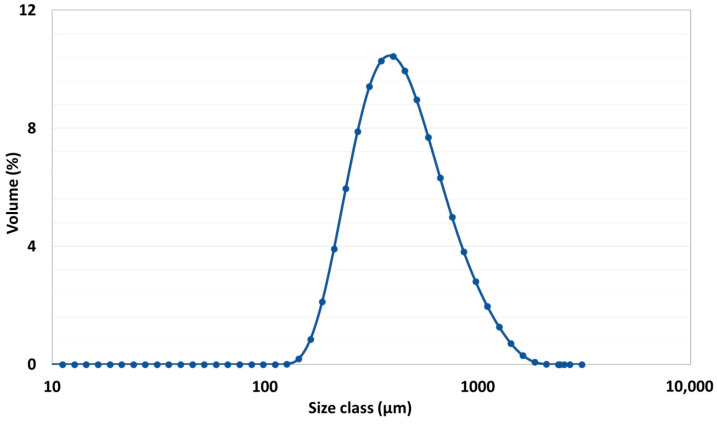
Average final particle size distribution in the 0.5 L vessel at 600 rpm.

In the preceding experiment, the granules were sampled, and their shape was characterized via scanning electron microscopy (SEM) using an FEI QUANTA 250 FEG microscope (Hillsboro, OR, USA). The resulting SEM images of the granules obtained with an impeller speed of 600 rpm are presented in Figure 1, displaying the temporal sequence of granule formation (with the images captured at 0, 4, and 10 min). The MCC granules maintain their original spherical shape over time, supporting the hypothesis that the initial particle sphericity of the powder can be assumed in numerical studies. The findings are further supported by a particle size distribution analysis using a Mastersizer Malvern 3000 laser diffraction (Worcestershire, UK) device in aero mode. The parameters for the measurements were set as follows: an air pressure of 10^2^ kPa, a vibration intensity of 40%, and a hopper gap maintained at 1.5 mm to avoid triggering the granule fragmentation mechanism. The particle size analysis was performed based on Mie scattering theory, following a standardized protocol. To ensure the accuracy of the results, 10 readings were taken and averaged for each sample. The median particle size, represented as the mean diameter D50, was used as the primary size descriptor. The results are presented as size classes, weighted by the volume fractions. The particle size distribution is illustrated in Figure 2, which reveals a mean diameter D50 of 488 μm, indicating a monomodal, narrow distribution. These results form the basis for subsequent numerical tests, aimed at experimentally validating simulation data.

### 2.2. Numerical Study

#### 2.2.1. DEM Modeling

The DEM was employed as the numerical method in this study; its theoretical underpinnings were initially outlined by Cundall and Strack in 1979 [18]. The granular flux can be predicted by considering the motion of individual particles and their mutual interactions. The position and velocity of a given particle *i* are determined by the time solution of Newton’s second law for the translational and rotational motions of this single solid particle. This process involves computing forces that act on each particle due to gravity, contact with other particles, and external forces, providing a dynamic simulation of particle behavior.

The incorporation of a contact model is essential for describing the behavior of different elements upon contact. The Hertz–Mindlin model was employed with the JKR model [22] in this study to simulate a cohesive contact model that takes into account the influence of van der Waals forces in the contact zone and allows for the modeling of strongly adhesive systems, such as cohesive dry powders. The JKR normal force is contingent on the overlap, δ, and the surface energy, γ (interaction element), as outlined in Equations (1) and (2):

(1) $$F_{JKR}=-4\sqrt{\pi \gamma E^{*}}a^{\frac{3}{2}}+\frac{4E^{*}}{3R^{*}}a^{3}$$

(2) $$\delta =\frac{a^{2}}{R^{*}}-\sqrt{4\pi \gamma a/E^{*}}$$

where E* and R* are the equivalent Young’s modulus and radius, respectively, defined in the Hertz–Mindlin contact model. If the surface energy is null, the JKR force returns to the normal Hertz–Mindlin force expression, given in Equation (3):

(3) $$F_{Hertz}=\frac{4}{3}E^{*}\sqrt{R^{*}}\delta_{n}^{\frac{3}{2}}$$

These contact models are essential for accurately predicting how particles will be bonded or separated during the granulation process, thus influencing the final product characteristics such as size distribution and density.

A calibration was required to ascertain the values of the particle/particle friction coefficient in the Hertz–Mindlin model and the surface energy (*γ*) in the JKR model. The friction coefficients were initially determined based on experimental data from Mesnier et al., 2019 [19] and subsequently refined through iterative simulation adjustments. For surface energy, settings were adjusted through a parametric study during the granulation process, which identified 1.7 J/m^2^ as the optimal value to prevent segregation and excessive agglomeration. The mechanical properties of the MCC powder are presented in Table 1. The calibration was conducted using experimental measurements of the angle of repose. A detailed description and discussion of the entire model, the coefficient values, and the calibration approach can be found in a previous study [29].

#### 2.2.2. Numerical High-Shear Wet Granulation

**Figure 3:**
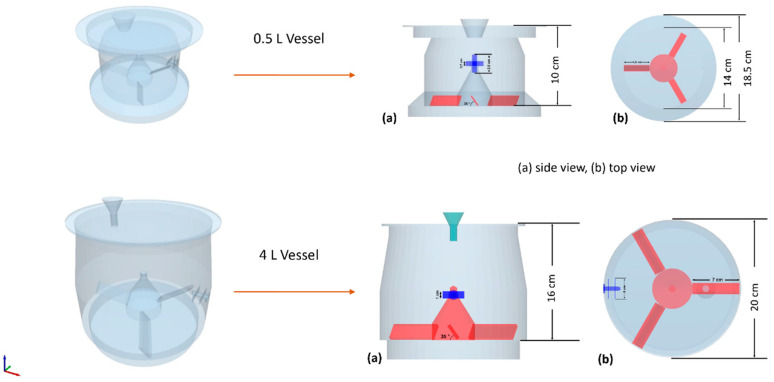
Three-dimensional representation of the numerical granulator on 0.5 and 4 L scales.

The simulations were conducted on a DELL T7910 workstation equipped with dual Intel Xeon E5-2660v3 processors, each featuring 10 cores, using the Academic EDEM 2020.1 commercial software. The initial phase of the project involved the design of the granulator vessel—incorporating the lid, impeller, and cross-blade chopper—using FreeCAD V.0.18, an open-source software licensed under LGPLv2+. Thereafter, the three-dimensional geometry of the granulator was exported and refined in EDEM simulation software. Figure 3 illustrates the two aforementioned numerical geometries of the granulators, presenting both side and top views.

**Figure 4:**
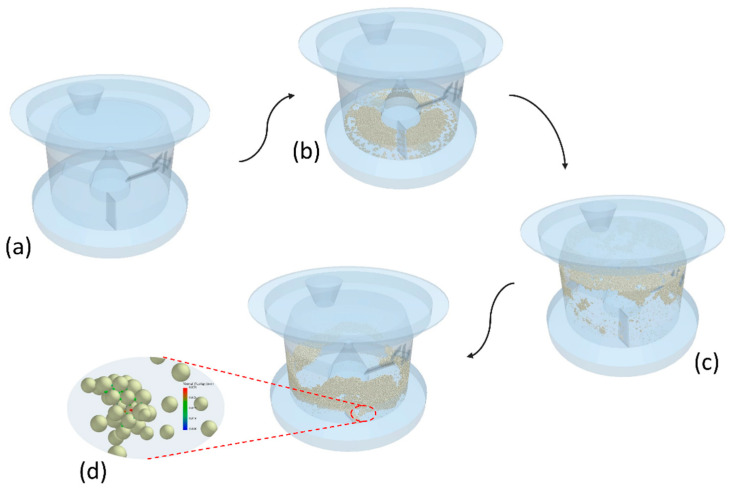
Numerical steps of granulation: (**a**) initial testing, (**b**) particle loading, (**c**) granulation, and (**d**) granule formation.

The process is divided into several stages to ensure a comprehensive evaluation and understanding of each phase: (a) initial testing of the agitation blades under no-load conditions, conducted to ensure mechanical integrity and operational readiness; (b) filling with particles, involving the systematic introduction of raw materials into the granulator; (c) granulation, engaging both the impeller and cross-blade chopper and conducted precisely according to the established experimental protocol to ensure consistency; and (d) granule formation. During the final stage, the JKR model is activated to simulate the cohesive forces between particles, thereby facilitating the formation of granules. The model’s effectiveness in capturing the nuanced dynamics of particle interaction is critical for predicting the outcome of the granulation process. Figure 4 presents this progression visually, underscoring the continuous and progressive development of granules.

#### 2.2.3. Posttreatment Developed Tool

**Figure 5:**
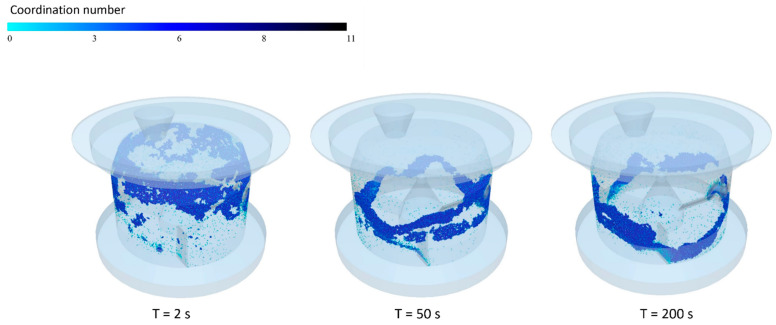
Representative image data from EDEM, depicting the variation of particle coordination numbers over time.

The granulation process, as observed through a series of simulations via our EDEM software, was initially found to occur in two stages. Initially, EDEM facilitated direct access to data collected in the numerical granulations over time, including relative speeds, collision counts, contact numbers, and coordination indices (an example is shown in Figure 5). These data were processed and analyzed within EDEM. However, the particle size distributions were only accessible prior to granulation (starting individual particle sizes only). The inability to track the growth kinetics of the granules was due to the simulation tool only accounting for the individual interactions of contacting mono-particles and not the properties of a cluster of agglomerated particles. This was problematic, as quantifying the size of the resulting granules was necessary to verify the numerical model and validate it experimentally. In addressing the challenges of quantifying granule sizes from DEM simulations, an advanced artificial intelligence (AI)-based approach was used to implement a solution through a custom-developed Python (Spyder v.5) procedure, facilitated by the EDEMpy package. This procedure enhances conventional data processing capabilities by intelligently sorting and analyzing the interactions between particles within the simulations.

Moreover, EDEMpy AI automates many of the processes involved in the traditional DEM-only models, which require significant user input for setup and analysis. This results in a significant reduction in the time required for model calibration and iterative testing. This automation facilitates rapid prototyping and testing of different process configurations. In contrast, PBM are effective in predicting overall particle size distributions, however, they do not readily incorporate dynamic changes from actual processing conditions or the impact of discrete particle interactions as EDEMpy AI can.

The algorithm dynamically classifies pairs of particles into two distinct groups: those in permanent contact, forming agglomerates; and those in transient contact due to collisions. This distinction is crucial, as it allows the tool to track and predict the aggregation behavior of particles over time, a significant advancement over traditional static analytical methods.

The use of this tool in this context involves a complex algorithm that not only retrieves and processes raw contact data from the simulations but also predicts the formation of particle clusters via temporal analysis. The Python-based AI process calculates the smallest envelope volume that encompasses all particles within a cluster, accurately determines the cluster mass and center of mass, and links these parameters to individual particle identifiers.

The ability to accurately determine particle sizes through volume and mass calculations underlines the accuracy of this method. The volumetric diameter is derived from the calculated shell volume using the formula $d_{v}={\left(\frac{6V}{\pi }\right)}^{1/3}$, where *V* is the shell volume containing all agglomerated particles.

**Figure 6:**
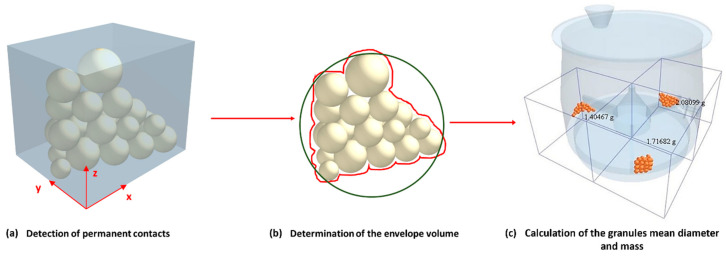
Representative steps for granule size determination in a 4 L vessel: (**a**) identification of permanent contacts, (**b**) calculation of envelope volume, and (**c**) determination of mean diameter and mass of granules.

This approach not only refines the data analysis but also streamlines the process, significantly reducing computational time requirements compared to traditional methods. This tool was validated on a sample of 100 particles. Initially, the particle count for each cluster was determined manually, and subsequently, these results were cross-verified using the AI tool. Both methods converge on identical outcomes, as presented in Table 2: cluster 1 consists of 40 particles weighing 2.081 g, cluster 2 comprises 33 particles weighing 1.717 g, and cluster 3 contains 27 particles weighing 1.405 g, as illustrated in Figure 6. The validation of this tool reveals its ability to reconcile manual counts and analytical results, demonstrating its reliability and effectiveness in improving granulation research.

## 3. Results and Discussion

The present section assesses the precision of the numerical model by comparing its predictions with experimental data obtained under two distinct operational conditions: impeller velocities of 600 rpm (with and without a chopper) and 900 rpm. These conditions have been discussed in Section 2.1.

### 3.1. Particle Generation and Numerical Limitations

In the simulation, the 0.5 L granulator vessel was filled to 25% of its volume, resulting in a simulated powder output of 62 g. The simulation generated 385,078 particles, with an initial size distribution ranging from 258 to 822 μm and a mean diameter of 592 μm. This distribution closely matches the experimental particle size distribution, with a standard deviation of 95 μm, suggesting a high degree of numerical accuracy in particle representation. The numerical model requires dividing the granulator’s geometry into cells for the effective detection of particle–particle contacts. The cell dimensions were strategically calculated based on the radius of the smallest particle, with each cell measuring 2.5 times the radius from the smallest to the largest particle. Importantly, reducing the particle size leads to an increase in the number of cells, significantly elevating the computational time. Therefore, a balance was sought between the smallest feasible particle size and the maximum number of contact detection cells, ensuring the computational viability of the simulation.

**Figure 7:**
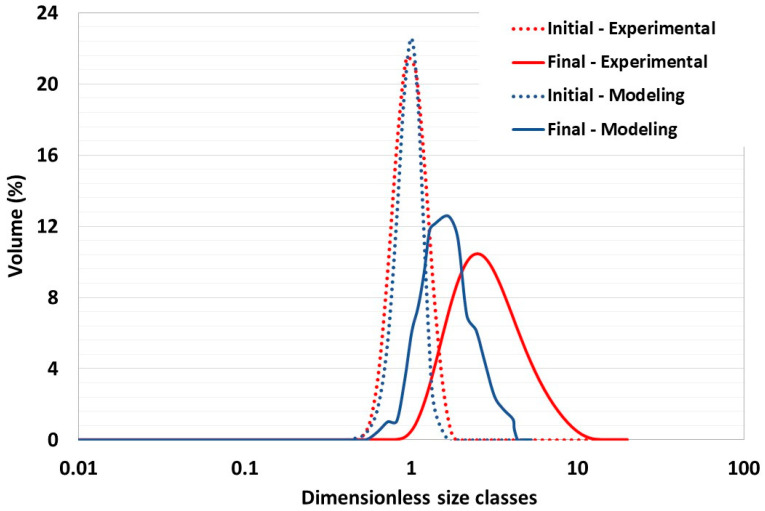
Comparison of experimental and numerical initial and final particle size distributions at 600 rpm. Dashed lines: initial PSD, continuous lines: final PSD, red lines: experimental results, blue lines: numerical results.

While adjustments were made to the initial particle size to accommodate computational limitations, the model still successfully captured the granule volume distribution of the experimental data, albeit with slight underrepresentation in some classes. The distribution is presented in a semilogarithmic format, which reflects the model’s effectiveness. Of particular note is that the mean diameter of the particles increases proportionately, attaining a threefold increase in experimental tests and a twofold increase in simulations. This outcome is indicative of the model’s precision in maintaining accurate scaling between the initial powder and the granules, as shown in Figure 7. The results are indicative of the model’s robust ability to predict general trends and provide qualitative insights into the granulation process.

In order to analyze the accuracy of our numerical model throughout the simulation, a Root Mean Square Error of Prediction (RMSEP) analysis was performed, comparing interpolated numerical data with experimental measurements. The results of the analysis showed an RMSEP value of 0.979 for the initial particle size distribution, indicating a high degree of accuracy in the model predictions at the initial stage. For the final particle size distribution, the RMSEP was calculated to be 2.219. Although this value is a little higher, it still reflects a reasonable predictive performance given the complexity and potential variability in the experimental setup and simulation parameters.

The RMSEP analysis supports the ability of the model to accurately reproduce particle size distributions, demonstrating good early-stage predictions and the ability to maintain trend accuracy to the end of the simulation. This statistical validation highlights the practical utility of the model in simulating granulation processes, providing insights for operational optimization and improved particle representation.

### 3.2. Parametric Analysis

#### 3.2.1. Influence of Impeller Velocity

**Figure 8:**
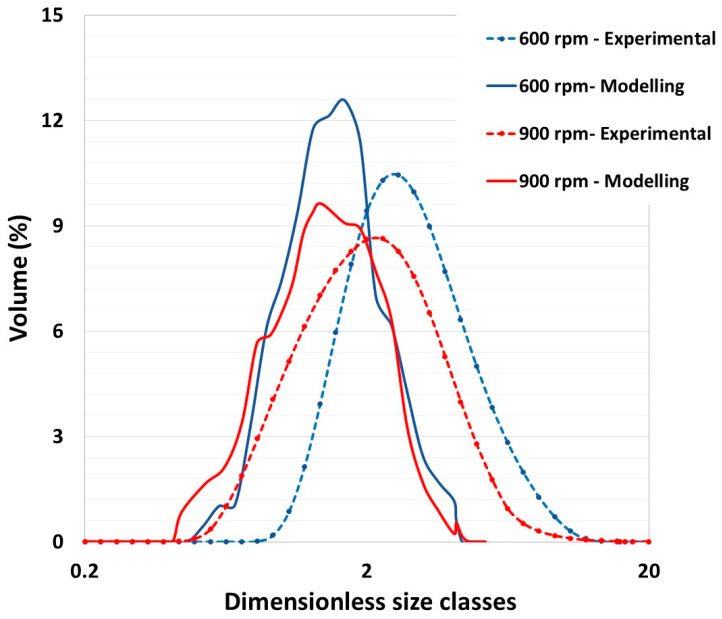
Comparison of experimental and numerical particle/granule size distributions at 600 and 900 rpm.

Figure 8 compares the experimental data and numerical results for two impeller velocities (600 and 900 rpm), utilizing the methodologies outlined in Section 2. The granule size distributions, derived from 62 g of the initial powder, were plotted in dimensionless size classes (normed mode) on a logarithmic scale weighted by volume fractions. The blue and red lines represent the final distributions after granulation at impeller velocities of 600 and 900 rpm, respectively. The dashed lines represent the experimental data, and the solid lines represent the model predictions.

A statistical analysis of the results is provided in Table 3. Despite the presence of quantitative disparities, presumably attributable to an underestimated surface energy value in the simulations, the numerical predictions demonstrate a general congruence with the experimental outcomes. It is noteworthy that the impeller’s shear stress induces a reduction in the mean diameter of the granules. However, the breadth of the distribution remains largely unaffected by the impeller speed, indicating that the impeller velocity primarily influences the granule size rather than the distribution spread.

The findings emphasize the model’s capacity to predict alterations in granule size, contingent on intrinsic properties such as the particle size and distribution, in conjunction with operational parameters such as the impeller speed. The comparison elucidates the model’s capabilities and limitations, guiding further refinements and validations of the numerical approach to better replicate experimental outcomes.

One should note that in the literature, this parameter is debated at the experimental level. Saleh et al. showed that an increase in the impeller velocity leads to an increase in the average diameter of the granules [1], whereas Ramaker et al. [33] revealed similar conclusions to those of our current study.

#### 3.2.2. Influence of the Chopper

**Figure 9:**
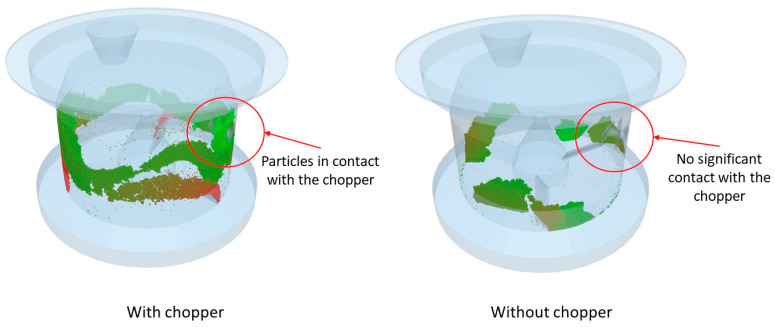
State of the particles in the 0.5 L vessel with and without a chopper.

**Figure 10:**
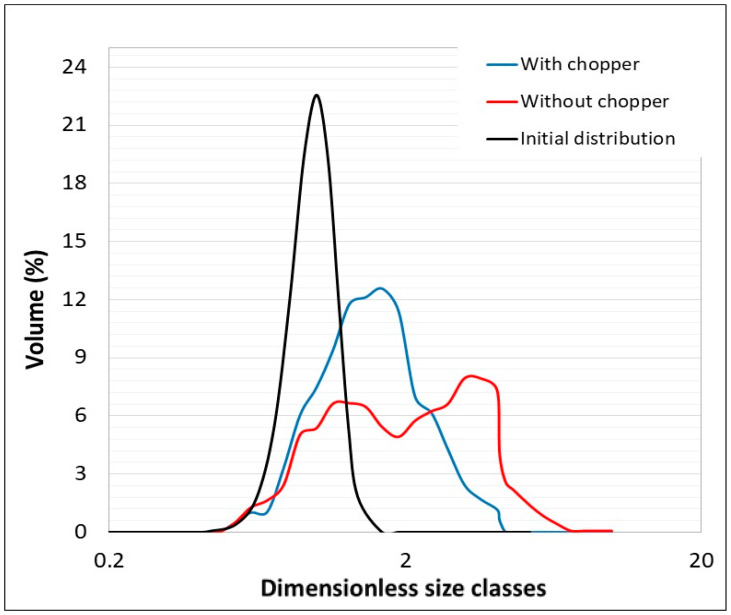
Comparison of numerical particle size distributions with and without a chopper.

A numerical investigation was conducted into the effect of the chopper while maintaining the impeller velocity at 600 rpm. Figure 9 compares the simulation outcomes in scenarios with and without the chopper operating at the same impeller speed. In accordance with our expectations and as substantiated in prior experimental research [1], the absence of a chopper leads to a broader particle size distribution, as presented in Figure 10. This phenomenon is attributed to the chopper’s function of fragmenting the larger agglomerates that tend to form during the granulation process.

Further analysis of the particle velocities over time reveals that the inclusion of a chopper significantly increases the average particle velocity, with the measurements showing a tenfold increase (from 1.28 m/s without the chopper to 10.14 m/s with the chopper). The higher velocity facilitates more frequent and forceful collisions between particles, effectively leading to the disintegration of larger agglomerates. This dynamic underpins the chopper’s critical function in enhancing the uniformity of the particle size distribution during the granulation process.

### 3.3. Numerical and Experimental Correlation

Notwithstanding the computational limitations that necessitated an adjustment of the particle size for the simulations, the numerical model exhibited remarkable accuracy in capturing key experimental trends, underscoring its robustness and adaptability. Specifically, the simulated volume distribution of granules aligns closely with the experimental data, presented in a semilogarithmic format. This close correspondence is maintained even though some particle size classes are slightly underrepresented due to the increased simulation particle size. The capacity of the model to accurately replicate the experimental distribution, despite the computational adjustments, reveals its precision and the efficacy of the underlying algorithms. This validates the model’s ability to reliably simulate the granulation process, thereby providing a robust foundation for gaining insights into the dynamics within the granulator.

The mean particle diameter increases consistently between the initial and final states in both the experimental tests and simulations, exhibiting a 1.8-fold increase in the experiments and a 2-fold increase in the simulations. This indicates that the model successfully captures the fundamental dynamics of particle growth during the granulation process while maintaining a proportional scaling reflective of real-world behavior. The model’s capacity to mirror these trends, even under the scaled-up particle sizes required for computational feasibility, highlights its potential for predictive modeling and simulation accuracy.

Notably, the model retains the ability to accommodate the impact of operational variables, such as impeller velocity, on the outcomes of the granulation process. This aspect of the model’s performance is particularly noteworthy, as it validates the model’s applicability in simulating real-world manufacturing environments under different operational settings. The capacity to extrapolate the effects of varying impeller velocities to other process conditions not only enhances the model’s utility but also supports its use in process optimization and scale-up studies. The incorporation of the chopper is particularly beneficial, as it effectively reduces the size of larger agglomerates, thereby promoting a more uniform particle size distribution and enhancing the overall efficiency and quality of the granulation output.

The discussion presented here emphasizes not only the success of the model in replicating experimental results but also the sophistication and scientific merit of the simulation approach. The positive implications of these results are significant in that they not only affirm the model’s accuracy and reliability in simulating complex processes but also open avenues for further improvements. These findings motivate the ongoing refinement of the simulation parameters and advocate for the development of more advanced computational techniques with AI tools for data processing to reduce resource demands without compromising the quality and accuracy of the results.

## 4. Conclusions

This paper presents the development and implementation of a numerical DEM model to simulate the high-shear granulation process. The study focuses on elucidating the complex interactions between the intrinsic characteristics of the powder, the operational parameters of the granulator, and the dynamic evolution of the quality of the produced granules. The model comprehensively accounts for the mechanical properties of particles, the dynamic processes linked to mixing, and the critical cohesion forces between particles.

A significant advancement in this research is attributable to the commercial software EDEM, which provided detailed insights into velocity profiles, cohesion forces, and coordination number changes. Nevertheless, the complexity of directly interpreting these data for experimental validation posed considerable challenges. To address this, a post-processing tool was developed using the EDEMpy AI tool, a Python-based extension, enabling the efficient discernment and categorization of particle contacts, with the subsequent isolation of permanent contacts indicative of agglomerate formation and the calculation of their volumes. The efficacy of this code has been thoroughly validated via a combination of numerical consistency checks and manual counting on a select number of particles. Further, the granule growth observed experimentally was compared with that predicted numerically in a 0.5 L granulator. This demonstrated the efficacy of the numerical particle size distribution in reproducing a Gaussian distribution when represented in a semilogarithmic scale, underscoring the importance of statistically representative particle counts. The growth ratios between the initial powder and the resulting granules, as well as the spread of the particle size distributions, were consistent across both the experimental and numerical platforms. This confirms the model’s predictive accuracy regarding the effects of impeller velocity.

These findings not only validate the model’s effectiveness but also highlight the potential of the EDEMpy AI tool to enhance one’s understanding and control of granulation processes. The integration of such advanced tools enables (1) secure scale-up processes, (2) the acquisition of temporal information complementary to torque measurements on the agitator shaft, and (3) precise predictions of the endpoint of wet granulation. From a practical perspective, the implications of integrating advanced simulation tools into pharmaceutical operations are far reaching, promising significant improvements in process optimization, intensification, cost efficiency, and product quality. This study establishes a precedent for the use of sophisticated simulation tools in pharmaceutics, paving the way for more efficient, scalable, and precise production methods.

From a future perspective, to enhance the robustness and applicability of the numerical DEM model in high-shear granulation, it is recommended to incorporate real-time monitoring technologies into experimental tests. Utilizing near-infrared spectroscopy (NIR) coupled with artificial intelligence (AI) routines for advanced data processing can provide immediate insights into the granulation process, allowing for real-time adjustments and optimization. This integration can significantly improve process control, ensuring consistency and quality in granule production. Simultaneously, there is a pressing need to expand the scope of numerical studies by adopting advanced multi-scale modeling approaches. These approaches, enhanced by AI, can bridge the gap between microscopic particle interactions and macroscopic process dynamics, offering a comprehensive understanding of the granulation process at all levels. Such advancements could help improve the predictive accuracy of the models and facilitate more effective scale-up and transferability of laboratory results to full-scale production environments.

## Figures and Tables

**Table 1:** Mechanical properties of microcrystalline cellulose (MCC).

Material Properties	Units	MCC
Poisson’s ratio (ν)	[-]	0.25
Density (ρ)	[kg/m^3^]	460
Young’s modulus (E)	[GPa]	8.67
Shear modulus (G)	[GPa]	3.47
Surface energy (γ)	[J/m^2^]	1.7
Particle–particle coefficients		
Coefficient of static friction	[-]	0.40
Coefficient of restitution	[-]	0.35
Coefficient of rolling friction	[-]	0.01
Particle geometry coefficients		
Coefficient of restitution	[-]	0.50
Coefficient of static friction	[-]	0.45
Coefficient of rolling friction	[-]	0.15

**Table 2:** Number of particles per cluster, with the corresponding mass calculations for each mode.

	Number of Particles	Mass Calculation [g]		
		Manual Count	EDEM	EDEMpy AI Tool
Cluster 1	40	2.081	2.081	2.081
Cluster 2	33	1.717	1.717	1.717
Cluster 3	27	1.405	1.405	1.405

**Table 3:** Statistical analysis of the particles: dimensionless mean diameter and standard deviation after granulation at 600 and 900 rpm.

	PSD at 600 rpm (μm)		PSD at 900 rpm (μm)	
	Mean Average	Standard Deviation	Mean Average	Standard Deviation
Experimental	3.16	1.64	2.35	1.36
Modeling	1.66	0.71	1.50	0.68

PSD: particle size distribution.

## Data Availability

The original contributions presented in this study are included in the article. Further inquiries can be directed to the corresponding authors.

## Glossary

AI	Artificial intelligence
DEM	Discrete element method
HSG	High shear granulator
JKR	Johnson–Kendall–Roberts
MCC	Microcrystalline cellulose
NIR	Near-infrared spectroscopy
PBM	Population balance modeling
PSD	Particle size distribution
PVP	Polyvinylpyrrolidone
RMSEP	Root Mean Square Error of Prediction
SEM	Scanning electron microscopy
